# Exploring the Prognostic Role of Trop-2, CD47, and CD163 Expression Levels on Survival Outcomes in Patients with Triple-Negative Breast Cancer

**DOI:** 10.3390/diagnostics15020232

**Published:** 2025-01-20

**Authors:** Ramazan Oguz Yüceer, Sedanur Aydın, Iclal Gelir, Tulay Koc, Ersin Tuncer, Mahmut Ucar

**Affiliations:** 1 Department of Pathology, Sivas Cumhuriyet University School of Medicine, Sivas 58140, Turkey; ksedanur@gmail.com (S.A.); tkoc@cumhuriyet.edu.tr (T.K.); etuncer@cumhuriyet.edu.tr (E.T.); 2 Sivas Cumhuriyet University School of Medicine, Sivas 58140, Turkey; iclallgelirr@gmail.com; 3 Department of Medical Oncology, Sivas Cumhuriyet University School of Medicine, Sivas 58140, Turkey; dr.mahmutucar@gmail.com

**Keywords:** breast cancer, triple-negative, Trop-2, CD47, CD163, tumor-associated macrophages, biomarker, immunotherapy, prognosis, survival

## Abstract

**Background**: This study evaluated the prognostic impact of Trop-2, CD47, and CD163 expression on clinical outcomes in triple-negative breast cancer (TNBC) and investigated their interactions with tumor progression. **Methods**: A retrospective cohort of 92 patients with TNBC was analyzed. The expression scores for Trop-2, CD47, and CD163 were categorized as negative/low (0–3 points) or high (4–6 points). The primary endpoint was overall survival (OS). **Results**: The median age of the cohort was 50 years old. High Trop-2 expression was observed in 55.4% of the patients and was significantly associated with advanced disease stage (*p* < 0.001). High CD47 expression (44.6%) was correlated with advanced stage (*p* = 0.044), whereas high CD163 expression (45.7%) was associated with advanced stage (*p* = 0.021), absence of comorbidities (*p* = 0.022), and lower pT stage (*p* = 0.023). Moderate positive correlations were found between Trop-2 and CD47 (*p* = 0.022), Trop-2 and CD163 (*p* = 0.037), and CD47 and CD163 (*p* < 0.001), respectively. Kaplan–Meier survival analysis revealed that patients with low Trop-2 expression exhibited significantly prolonged OS (*p* = 0.021) and progression-free survival (PFS) (*p* = 0.026) compared to those with high Trop-2 expression. Univariate and multivariate analyses revealed significant associations between OS and PFS for Trop-2, lymphovascular invasion, and BRCA status. **Conclusions**: Trop-2 expression is a significant prognostic factor for TNBC and is correlated with worse outcomes. Although CD47 and CD163 showed trends for poorer prognosis, their significance was not confirmed. These findings offer promising prospects for future studies on combined antibody–drug conjugates (ADCs), as they may present opportunities to address multiple resistance mechanisms in the management of TNBC and enhance clinical outcomes.

## 1. Introduction

Breast cancer (BC) is the most frequently diagnosed malignancy among women worldwide and is a major contributor to cancer-related mortality [1]. Although survival rates have significantly improved with advancements in early detection and treatment, advanced-stage disease remains associated with poor prognosis and aggressive clinical progression [2]. Triple-negative breast cancer (TNBC), comprising approximately 10–15% of all BC cases, is characterized by a biologically heterogeneous profile [2,3]. The absence of hormone receptors and HER2 expression in TNBC limits the availability of targeted therapeutic options [4]. This subtype typically presents at a younger age and is distinguished by its aggressive behavior, marked by a high metastatic potential and reduced disease-free survival [2]. The mortality rates for TNBC were notably higher than those observed for the other BC subtypes [4]. Although local therapies, including surgery and radiotherapy, play an important role, chemotherapy remains the primary systemic treatment modality for TNBC. Emerging therapeutic approaches, such as immunotherapy [5] and poly (ADP-ribose) polymerase (PARP) inhibitors [6], have demonstrated encouraging outcomes in recent years. Nonetheless, the identification and application of prognostic and predictive biomarkers are increasingly pivotal for tailoring treatment strategies and enhancing clinical management. Consequently, research efforts focusing on the biological stratification of TNBC are critical for advancing personalized treatments and improving patient outcomes.

Trophoblast cell-surface antigen-2 (Trop-2) is a 35 kDa transmembrane glycoprotein encoded by the Tacstd2 gene [7]. Initially identified in trophoblast cells, Trop-2 is involved in intracellular calcium signaling, and has been implicated in cancer pathophysiology. Specifically, it plays a pivotal role in key processes of the MAPK/PI3K/AKT signaling pathway, contributing to tumor proliferation, invasion, and metastasis [8,9]. Trop-2 expression has been observed in various tissues, including the skin, breast, and cervix, with elevated levels often correlating with poorer clinical outcomes and reduced survival across multiple cancer types [10,11,12,13]. In the context of rapidly evolving treatment paradigms, in which combination regimens integrating immunotherapy or targeted agents with standard chemotherapy are increasingly utilized to address aggressive tumor behavior and resistance mechanisms, antibody–drug conjugates (ADCs) have emerged as a promising therapeutic strategy. Notably, sacituzumab govitecan, an ADC targeting Trop-2, has demonstrated substantial clinical efficacy in clinical trials. Following its FDA approval, it has been incorporated into standard treatment protocols for malignancies such as TNBC [14] and urothelial carcinoma [15], offering significant clinical benefits.

Tumor-associated macrophages (TAMs) are pivotal components of the tumor microenvironment (TME) that significantly influence cancer progression, immune suppression, and metastasis processes [16,17]. Among the key molecules that reflect TAMs’ activity and function, CD47 and CD163 stand out [18]. CD47, often referred to as the “don’t eat me” signal, is an immune checkpoint molecule that allows cancer cells to evade phagocytosis [19,20]. Elevated CD47 expression across various cancer types has been linked to enhanced immune evasion and unfavorable prognostic outcomes [21,22,23]. Conversely, CD163, a surface receptor characteristic of anti-inflammatory M2 macrophages, contributes to the establishment of an immunosuppressive microenvironment that facilitates tumor progression [24]. High levels of CD163 expression are associated with increased tumor cell proliferation, angiogenesis, and metastatic spread [25,26]. Recent studies have shown that the upregulation of both CD47 and CD163 is correlated with poor clinical outcomes and reduced survival rates [27]. Emerging therapeutic strategies targeting these molecules aim to mitigate the pro-tumoral activities of TAMs while potentiating the anti-tumor response of the immune system. Specifically, anti-CD47 therapies and interventions designed to modulate the phenotypic transformation of TAMs represent promising approaches to improve the effectiveness of immunotherapy.

From an immunotherapeutic perspective, the interplay between Trop-2 and TAMs’ activity within the TME is particularly significant [28]. The overexpression of Trop-2 is hypothesized to amplify the pro-tumoral functions of TAMs by promoting their polarization toward the M2 phenotype. This process is mediated through an enhanced secretion of cytokines, including IL-10 and TGF-β, which fosters an inflammatory and immunosuppressive microenvironment [28]. Growth factors and metalloproteinases released by TAMs can activate Trop-2-mediated signaling pathways, thereby increasing the invasive and metastatic capacities of tumor cells [29]. Preclinical studies have suggested that Trop-2 inhibition could synergize with CD47 blockade by augmenting macrophage-mediated phagocytosis [30]. Furthermore, the Trop-2 activation of the PI3K/AKT and MAPK signaling pathways reinforces TAM integration into the TME, further suppressing immune responses and driving tumor progression [31]. This reciprocal interaction between Trop-2 and TAMs contributes to more aggressive tumor phenotypes and is associated with poor clinical outcomes. Combined therapeutic strategies targeting Trop-2 and TAM-related activities such as CD47 and CD163 offer a promising approach to disrupt this deleterious synergy. Such strategies have the potential to broaden treatment options and improve cancer management by simultaneously inhibiting tumor growth and mitigating immunosuppression. These dual-target approaches merit further investigation in clinical research, representing an innovative avenue for advancing immunotherapeutic strategies.

The main objective of this study was to investigate the relationship between the expression levels of Trop-2, CD47, and CD163, as assessed by immunohistochemistry in tumor tissue, and clinical outcomes and survival in TNBC, which is characterized by an aggressive clinical course and poor prognosis. In doing so, this research provides potential contributions to the oncology literature for future ADC studies, offering insights that could expand treatment options, address resistance mechanisms, and improve clinical outcomes.

## 2. Materials and Methods

### 2.1. Design, Selection of the Patients and Data Collection

This study was designed using retrospective data from 110 patients diagnosed with TNBC, histopathologically confirmed according to the World Health Organization (WHO) Classification of Breast Tumors, 4th Edition [3], who were treated and followed up between January 2014 and December 2022 at the Oncology Department of Sivas Cumhuriyet University Health Services Application and Research Hospital. After excluding 18 patients whose clinical data were lost during follow-up or whose tumor tissue was insufficient for immunohistochemical analysis, the final analysis included 92 patients selected in accordance with the study design.

In addition to the absence of an estrogen receptor, a progesterone receptor, and HER2 expression, which define TNBC, tumor tissue samples were evaluated for androgen receptor status, tumor grade, lymphovascular invasion, and Ki67 proliferation index in accordance with the 2018 protocol of the College of American Pathologists (CAP). Clinical and sociodemographic data were retrieved from oncology archive records and the hospital information system, including patient age, menopausal status (categorized as premenopausal for age <50 years and postmenopausal for age ≥50 years), pT stage and nodal involvement classified by the 8th Edition of the American Joint Committee on Cancer (AJCC) TNM classification system, germline BRCA mutation status, Eastern Cooperative Oncology Group performance status (ECOG PS), presence of comorbidities, disease burden, and survival.

### 2.2. Immunohistochemical Study and Evaluation of Trop-2, CD47, and CD163 Expressions

The tumors of patients diagnosed with TNBC were re-examined using hematoxylin and eosin (H&E)-stained sections, and paraffin blocks containing tumor tissue without hemorrhage or necrosis were selected. From these blocks, 3-micron-thick sections were cut and transferred to adhesive-coated slides. The sections were then incubated with the following primary antibodies: Trop-2 (mouse monoclonal antibody, Clone B-9, 1:100 dilution, Santa Cruz Biotechnology, Paso Robles, CA, USA), CD47 (rabbit monoclonal antibody, EPR21794, 1:2000 dilution, Abcam, Cambridge, UK), and CD163 (mouse monoclonal antibody, MRQ-26, 1:100 dilution, Roche Ventana, Tucson, AZ, USA). To validate the staining, tissue samples were processed alongside the positive control tissues: squamous epithelium for Trop-2 and tonsil tissue for CD47 and CD163. Negative control liver tissues for Trop-2, CD47, and CD163 were included in this study. All immunohistochemical staining was performed using a fully automated device (Roche Ventana Benchmark Ultra, Tucson, AZ, USA). The stained slides were then evaluated by two pathologists (R.O.Y. and S.D.A.), who were blinded to the clinical data, and both had expertise in breast cancer pathology.

**Figure 1:**
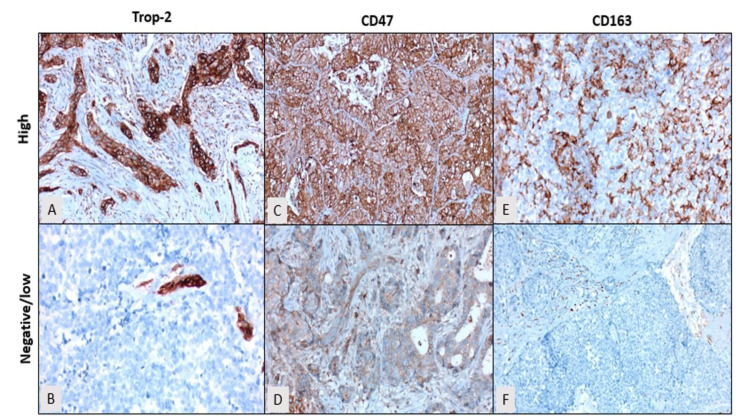
(**A**) Membranous high expression of Trop-2 in tumor cells. (**B**) Negative staining for Trop-2 in tumor cells and membranous high expression in mammary ductal cells. (**C**) Membranous high expression of CD47 in tumor cells. (**D**) Membranous low expression of CD47 in tumor cells. (**E**) Dot-like granular high expression of CD163 in peritumoral and intratumoral macrophages. (**F**) Negative expression of CD163 in peritumoral and intratumoral macrophages (DAB, ×200). DAB (diaminobenzidine).

For Trop-2, positive membranous and/or cytoplasmic staining was considered indicative of a positive expression. The intensity of expression was scored as follows: 0, none; 1, weak; 2, moderate; and 3, strong. The extent of staining in the tumor cells was graded as 0 = 0%, 1 = 1–19%, 2 = 20–50%, and 3 = >50% (Figure 1). The H-score is calculated by multiplying the intensity and extent scores. On the basis of the H-score, Trop-2 expression was categorized into two groups: negative/low (0–3 points), and high (4–6 points) [10]. For CD47, positive membranous and/or cytoplasmic staining was considered positive, whereas for CD163, positive cytoplasmic or dot-like granular staining was regarded as positive. The intensity of expression of both CD47 and CD163 was graded as follows: 0, none; 1, weak; 2, moderate; and 3, strong staining. The extent of staining was evaluated as follows: 0 = 0%, 1 = 1–19%, 2 = 20–50%, and 3 = >50%. H-scores for CD47 and CD163 were calculated in the same manner and classified into two groups based on the H-score: negative/low (0–3 points), and high (4–6 points) [20].

### 2.3. Follow-Up, Response Assessment, and Ethical Considerations

All patients diagnosed with TNBC were treated according to the standard treatment protocols established by the National Comprehensive Cancer Network (NCCN), according to the 8th Edition of the AJCC guidelines. Clinical responses were assessed and categorized according to the Response Evaluation Criteria in Solid Tumors (RECIST) guidelines (version 1.1). Progression-free survival (PFS) was defined as the time from initial diagnosis to disease progression, death, or the last follow-up. Overall survival (OS) was calculated as the time from histological diagnosis to death or the last follow-up. The primary outcome of interest was the OS.

This study was conducted in accordance with the Declaration of Helsinki, as adopted in 1964 and revised in 2013, with strict adherence to the ethical principles. The study protocol was comprehensively reviewed and approved by the Non-Interventional Clinical Research Ethics Committee of Sivas Cumhuriyet University on 16 May 2024 (Approval Number: 2024/05-30). Owing to the retrospective nature of the study, patient consent was not required. However, all data were anonymized to ensure patient confidentiality.

### 2.4. Statistical Analysis

Statistical analyses were conducted using Statistical Package for Social Sciences (SPSS) version 27 for Windows (IBM SPSS Inc., Chicago, IL, USA). The required sample size was determined using G-Power 3.1.9.7. Assuming an effect size of 0.50, a type I error rate of 0.05, and a power of 95%, it was calculated that 80 tissue samples would be sufficient for the study. The normality of the distribution of continuous data were assessed using either the Kolmogorov–Smirnov test or the Shapiro–Wilk test. Continuous variables with a normal distribution are presented as mean ± standard deviation, whereas those without a normal distribution are reported as medians (min–max). Categorical variables were compared using Pearson’s chi-square test, and Fisher’s exact test was applied when the expected values were insufficient. Overall survival (OS) and progression-free survival (PFS) were estimated using the Kaplan–Meier method, and survival differences between the groups were compared using the log-rank test. The median survival times are reported with 95% confidence intervals (CIs). For univariate and multivariate survival analyses, hazard ratios (HRs) and 95% CIs were calculated using the Cox proportional hazard regression model. Only variables found to be significant in the univariate analysis were included in the multivariate model. Statistical significance was defined as *p* < 0.05 for all analyses.

## 3. Results

### 3.1. Patient Characteristics

The study cohort comprised 92 patients with a median age of 50 years (range: 25–79 years), with 48.9% (45 patients) aged ≥50 years. Among them, 40.2% (37 patients) were premenopausal and 59.8% (55 patients) were postmenopausal. Comorbid conditions were identified in 35.9% (33 patients). Germline mutations in BRCA1 and BRCA2 were detected in 19.6% (18 patients). The tumor was located in the upper outer quadrant of the breast in 50% of the patients and was multifocal in 10.9% (10 patients). The ECOG PS scores were 0–1 in 82 patients and ≥2 in 10 patients. Tumor grade 3 was observed in 64.1% of the patients, pT stage 3–4 in 19.6%, nodal involvement in 53.3%, and distant metastases at diagnosis in 21.7%. Additionally, 53.3% of patients had stage 1–2 disease, whereas 46.7% had stage 3–4 disease. A Ki67 proliferation index greater than 50% was reported in 71.8% of cases. A detailed summary of the sociodemographic and clinicopathological characteristics of TNBC patients categorized according to Trop-2, CD47, and CD163 expression levels is presented in Table 1.

### 3.2. Trop-2, CD47, and CD163 Expressions

Among patients with TNBC, 44.6% (41 patients) exhibited negative or low Trop-2 expression, whereas 55.4% (51 patients) displayed high Trop-2 expression (Figure 1A,B). High Trop-2 expression was more frequently observed in postmenopausal patients, those without comorbidities, individuals with right-sided tumors, unifocal disease, low ECOG PS, high histologic grade, absence of lymphovascular invasion, lower pT stage, nodal involvement, and high Ki67 proliferation index. However, none of these associations were statistically significant (*p* > 0.05). Importantly, high Trop-2 expression was significantly associated with an advanced disease stage (*p* < 0.001) (Table 1).

In patients with TNBC, 55.4% (51 patients) demonstrated negative or low CD47 expression, whereas 44.6% (41 patients) showed high CD47 expression (Figure 1C,D). High CD47 expression was more frequently observed in postmenopausal women, patients without comorbidities, those with right-sided tumors, unifocal disease, low ECOG PS, high histologic grade, absence of lymphovascular invasion, lower pT stage, nodal involvement, and high Ki67 proliferation index. However, no statistically significant association was found between CD47 expression and these clinical factors (*p* > 0.05). Notably, high CD47 expression was significantly correlated with an advanced disease stage (*p* = 0.044) (Table 1).

In patients with TNBC, 54.3% (50 patients) exhibited negative or low CD163 expression, while 45.7% (42 patients) demonstrated high CD163 expression (Figure 1E,F). High CD163 expression was more frequently observed in postmenopausal women, those with right-sided tumors, unifocal disease, low ECOG PS, high histologic grade, absence of lymphovascular invasion, nodal involvement, and a high Ki67 proliferation index. Despite these trends, no statistically significant associations were found between CD163 expression and these clinical factors (*p* > 0.05). However, high CD163 expression was significantly associated with an advanced disease stage (*p* = 0.021), absence of comorbidities (*p* = 0.022), and low pT stage (*p* = 0.023) (Table 1).

A moderate positive correlation was observed between high Trop-2 expression and high CD47 expression (*p* = 0.022, *r* = 0.232), as well as between high Trop-2 expression and high CD163 expression (*p* = 0.037, *r* = 0.207). Furthermore, a significant positive correlation was found between high CD47 and CD163 expression (*p* < 0.001, *r* = 0.364) (Table 2).

### 3.3. Survival Analyses

In this cohort, with a median follow-up duration of 56.8 months, 26 patients (28.1%) died during the study period. The median OS was 76 months (95% CI, 66.0–85.9). Furthermore, 11 patients (11.2%) experienced disease recurrence during the follow-up. The median PFS for the entire cohort was 73 months (95% CI, 58.2–87.7).

**Figure 2:**
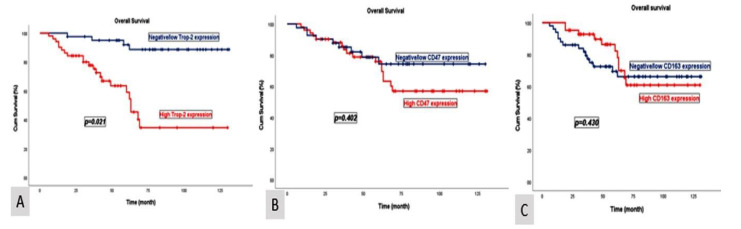
Kaplan–Meier curves illustrating the overall survival stratified by Trop-2 (**A**), CD47 (**B**), and CD163 (**C**) expression levels.

**Figure 3:**
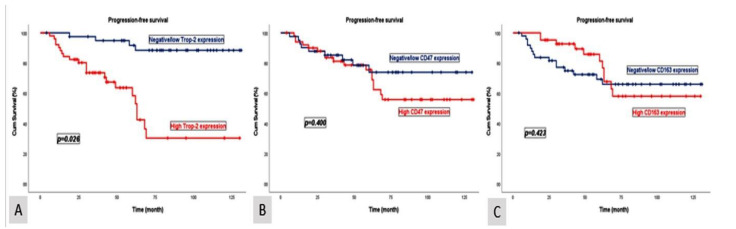
Kaplan–Meier curves illustrating the progression-free survival stratified by Trop-2 (**A**), CD47 (**B**), and CD163 (**C**) expression levels.

Patients with negative/low Trop-2 expression exhibited a median OS of 84 months (95% CI: 72.3–95.7) and a PFS of 80 months (95% CI: 65.7–97.6). In contrast, patients with high Trop-2 expression demonstrated a significantly shorter median OS of 54 months (95% CI: 45.6–62.4) and a PFS of 52 months (95% CI: 38.7–65.5). These differences were statistically significant for both OS (*p* = 0.021) and PFS (*p* = 0.026) (Figure 2 and Figure 3).

Patients with negative/low CD47 expression had a median OS of 84 months (95% CI: 61.6–106.3) and a PFS of 82 months (95% CI: 66.4–104.8). Conversely, patients with high CD47 expression demonstrated a median OS of 68 months (95% CI: 47.2–88.8) and a PFS of 65 months (95% CI: 42.1–87.9). Patients with negative/low CD163 expression exhibited a median OS of 79 months (95% CI: 66.0–92.1) and a PFS of 77 months (95% CI: 59.6–87.9). In contrast, patients with high CD163 expression had a median OS of 70 months (95% CI: 50.4–89.6) and a PFS of 65 months (95% CI: 40.2–89.8). The median OS and PFS outcomes compared according to CD47 and CD163 expression levels were clinically meaningful; however, the findings were not statistically significant. The *p*-values for the median OS and PFS analyses were as follows: CD47 (*p* = 0.402, *p* = 0.400) and CD163 (*p* = 0.430, *p* = 0.423), respectively (Figure 2 and Figure 3).

In univariate analysis using the Cox proportional hazards model, several factors were found to be significantly associated with OS. These included lymphovascular invasion, BRCA status, pT stage, distant metastasis, clinical stage, and Trop-2 expression (*p* < 0.05). Similarly, for PFS, univariate analysis revealed significant associations with lymphovascular invasion, BRCA status, tumor focality, pT stage, distant metastasis, clinical stage, and Trop-2 expression (*p* < 0.05) (Table 3). In multivariate analysis, significant associations with OS were identified for lymphovascular invasion, BRCA status, and Trop-2 expression (*p* < 0.05). For PFS, the multivariate analysis revealed that tumor focality, BRCA status, and Trop-2 expression were significantly associated with PFS (*p* < 0.05) (Table 4).

## 4. Discussion

TNBC continues to represent one of the most formidable subtypes of breast cancer and is characterized by its aggressive nature, high recurrence rates, and the absence of targeted hormonal or molecular therapies [2,4]. However, recent advancements in treatment strategies offer promise for improving patient outcomes. Immune checkpoint inhibitors targeting the PD-1/PD-L1 axis have emerged as a transformative approach in the field of immunotherapy [5,6]. Agents such as pembrolizumab and atezolizumab have demonstrated significant clinical benefits in patients with PD-L1-positive TNBC, leading to improved survival outcomes in select populations [5]. Furthermore, ADCs have demonstrated considerable potential. The first Trop-2-targeted ADC, sacituzumab govitecan, notably improved the survival of patients with advanced TNBC who had previously undergone chemotherapy [14]. Additionally, emerging immunomodulatory strategies targeting various components of the tumor microenvironment, including tumor-infiltrating lymphocytes (TILs), macrophages, and natural killer (NK) cells, are being actively explored. These strategies are being evaluated in combination with conventional therapies, with the aim of enhancing efficacy by leveraging the immune system’s ability to recognize and eradicate cancer cells. With ongoing clinical trials and biomarker-driven research, the integration of these novel therapies into clinical practice heralds a new era for TNBC treatment that emphasizes personalized medicine and improved survival outcomes.

Trop-2 has recently attracted considerable attention for its prognostic significance in cancer owing to its involvement in key signaling pathways such as MAPK, PI3K, and AKT, which are crucial for cell proliferation, survival, and metastasis [9,13]. Trop-2 overexpression has been associated with poor prognosis in various cancers, including breast cancer, by promoting tumor growth and metastasis through the activation of these pathways [8]. Trop-2’s interactions with the tumor microenvironment, particularly its modulation of immune cell behavior, play a pivotal role in cancer progression [28,31]. TAMs, often characterized by a high expression of CD47 and CD163, contribute to immune evasion and tumor progression [20]. Studies have shown that elevated Trop-2 expression, along with increased levels of CD47 and CD163, correlates with enhanced TAM activity and worse clinical outcomes, especially in TNBC [30]. Additionally, it has been proposed that Trop-2, via the PI3K/AKT and MAPK pathways, influences the tumor microenvironment by promoting the polarization of macrophages toward the M2 phenotype, which is linked to tumor-promoting processes, such as tissue remodeling, angiogenesis, and immune suppression [28,30,31]. The interplay between Trop-2 and immune checkpoint molecules, such as CD47, as well as the cross-talk between these pivotal signaling pathways, underscores a promising avenue for clinical research on combination therapies [30,31,32]. Such strategies have the potential to improve the clinical management of cancers, particularly TNBC, and enhance their therapeutic efficacy.

The findings of this study underscore the potential prognostic significance of Trop-2, CD47, and CD163 in the pathobiology of TNBC. Specifically, the results indicated that elevated Trop-2 expression correlates with adverse clinical outcomes, reinforcing its putative role in tumor progression and metastatic dissemination. Similarly, CD47 has been implicated in promoting tumor aggressiveness through its function in immune evasion, notably by inhibiting immune cell activity within the tumor microenvironment. High CD163 expression further highlights the immunosuppressive functions of TAMs, suggesting their pivotal role in fostering an immunosuppressive milieu, particularly in TNBC. Given the aggressive clinical course and limited therapeutic options for TNBC, these molecules warrant consideration as independent prognostic markers and as potential therapeutic targets. Furthermore, their inhibition in combination with immunotherapies may enhance the efficacy of current treatment modalities. While the study was constrained by a relatively small sample size, its findings establish a foundation for subsequent investigations. Prospective studies involving larger, more diverse cohorts are necessary to validate the prognostic value of these biomarkers and elucidate their potential utility in the clinical management of TNBC.

The prognostic implications of Trop-2 and TAM biomarkers have been explored extensively in prior research [32]. A review by Tong et al. examining the mechanisms of action and clinical applications of Trop-2-targeted ADCs in TNBC revealed that these agents facilitate immune reprogramming, modulate the tumor microenvironment, and enhance anti-tumor efficacy [32]. Subgroup analyses from the ASCENT trial demonstrated that the anti-Trop-2 ADC sacituzumab govitecan significantly improved clinical outcomes in TNBC patients, regardless of the BRCA1/2 mutation status [33]. Similarly, Rugo et al. reported that sacituzumab govitecan outperformed standard chemotherapy in improving the PFS of patients with HR+/HER2-negative metastatic breast cancer [34]. Additionally, the phase III TROPION-Lung01 trial showed that another anti-Trop-2 ADC, datopotamab deruxtecan, provided a PFS benefit over docetaxel in metastatic non-small cell lung cancer (NSCLC) [35]. In the context of TAM biomarkers, Dawoud et al. identified CD47 and CD163 as indicators of TAM activity and correlated their expression with poor prognosis in breast cancer [18]. Similarly, Imam et al. highlighted the negative prognostic impact of CD47 and CD163 expression in pancreatic neuroendocrine tumors [27]. The findings of our study align closely with these results, further supporting the prognostic significance of Trop-2- and TAM-associated biomarkers.

This study, which highlights the interactions between Trop-2 and TAM biomarkers in TNBC and their impact on clinical outcomes, had several limitations. The retrospective design, reliance on single-center data, and relatively small cohort size may limit the generalizability of our findings. Although the demographic distribution appears heterogeneous, the absence of a randomization process in patient selection further restricts the robustness of the study. Additionally, the inability to assess factors such as PD-L1 expression and microsatellite instability (MSI) in all cases, due to technical and financial constraints, diminishes the study’s overall power. Furthermore, the lack of globally standardized protocols for scoring the expression of these biomarkers in tumor tissues, coupled with variations in disease management strategies among clinicians in advanced-line treatment, may introduce variability in the interpretation of the results. Exploring the correlation between Trop-2 and other TAM markers, such as CD20, presents promising opportunities for future research. Incorporating functional studies to investigate its downstream interactions with pivotal signaling pathways, including PI3K, AKT, and MAPK, could yield valuable insights and substantially advance the understanding of its role in tumor biology. In addition, experimental studies are required to validate the functional role of Trop-2 in TNBC cell lines. Consequently, prospective multicenter studies are warranted to address these limitations and provide more definitive conclusions.

## 5. Conclusions

This study highlights the prognostic importance of Trop-2, CD47, and CD163 expression in TNBC, an aggressive and treatment-resistant subtype of breast cancer. High Trop-2 expression is significantly associated with advanced disease and poor clinical outcomes, including shorter OS and PFS. Although CD47 and CD163 showed trends toward worse outcomes, their prognostic value was not statistically significant. The interplay between these biomarkers suggests their role in modulating the TME, particularly through immune evasion and macrophage polarization. Trop-2𠌙s involvement in oncogenic pathways and the immunosuppressive effects of CD47 and CD163 underscore their potential as therapeutic targets. Although limited by its retrospective design and small sample size, this study provides a basis for further investigation. Larger prospective studies are needed to confirm these findings and to explore their integration into clinical practice to refine prognostic assessments and develop novel therapies for TNBC.

## Figures and Tables

**Table 1:** Sociodemographic and clinicopathological characteristics of patients stratified by Trop-2, CD47, and CD163 expression levels (all patients, *n* = 92).

Variables	Trop-2 Expression			CD47 Expression			CD163 Expression		
	Negative/Low	High	*p*	Negative/Low	High	*p*	Negative/Low	High	*p*
	*n*, (%)	*n*, (%)		*n*, (%)	*n*, (%)		*n*, (%)	*n*, (%)	
**Age**									
<50	20 (48.8)	27 (52.9)		24 (47.1)	23 (56.1)		24 (48.0)	23 (54.8)	
≥50	21 (51.2)	24 (47.1)	0.426	27 (52.9)	18 (43.9)	0.257	26 (52)	19 (47.1)	0.331
**Localization**									
Right	18 (43.9)	28 (54.9)		23 (45.1)	23 (56.1)		22 (44.0)	24 (57.1)	
Left	23 (56.1)	23 (45.1)	0.201	28 (54.9)	18 (43.9)	0.201	28 (56.0)	18 (42.9)	0.148
**Menopausal status**									
Premenopausal	16 (39)	21 (41.2)		20 (39.2)	17 (41.5)		17 (34.0)	20 (47.6)	
Postmenopausal	25 (61)	30 (58.8)	0.502	31 (60.8)	24 (58.5)	0.498	33 (66.0)	22 (52.4)	0.133
**ECOG PS**									
0–1	37 (90.2)	45 (88.2)		45 (88.2)	37 (90.2)		42 (84.0)	40 (95.2)	
≥2	4 (9.8)	6 (11.8)	0.516	6 (11.8)	4 (9.8)	0.516	8 (16.0)	2 (4.8)	0.080
**Comorbidity**									
No	24 (58.5)	35 (68.6)		30 (58.8)	29 (70.7)		27 (54.0)	32 (76.2)	
Yes	17 (41.5)	16 (31.4)	0.216	21 (41.2)	12 (29.3)	0.167	23 (46.0)	10 (23.8)	0.022
**Histological grade**									
1	2 (4.9)	1 (2)		2 (3.9)	1 (2.4)		2 (4.0)	1 (2.4)	
2	14 (34.1)	16 (31.4)		17 (33.3)	13 (31.7)		13 (26.0)	17 (40.5)	
3	25 (61)	34 (66.7)	0.459	32 (62.7)	27 (65.9)	0.693	35 (70.0)	24 (57.1)	0.332
**Lymphovascular invasion**									
Absent	22 (53.7)	29 (56.9)		30 (58.8)	21 (51.2)		26 (52.0)	25 (59.5)	
Present	19 (46.3)	22 (43.1)	0.461	21 (41.2)	20 (48.8)	0.302	24 (48.0)	17 (40.5)	0.304
**Tumor focality**									
Unifocal	39 (95.1)	43 (84.3)		45 (88.2)	37 (90.2)		43 (86.0)	39 (92.9)	
Multifocal	2 (4.9)	8 (15.7)	0.091	6 (11.8)	4 (9.8)	0.516	7 (14.0)	3 (7.1)	0.239
**pT stage**									
1–2	34 (82.9)	40 (78.4)		38 (74.5)	36 (87.8)		36 (72.0)	38 (90.5)	
3–4	7 (17.1)	11 (21.6)	0.394	13 (25.5)	5 (12.2)	0.090	14 (28.0)	4 (9.5)	0.023
**Nodal involvement**									
Absent	18 (43.9)	25 (49)		24 (47.1)	19 (46.3)		23 (46.0)	20 (47.6)	
Present	23 (56.1)	26 (51)	0.391	27 (52.9)	22 (53.7)	0.556	27 (54.0)	22 (52.4)	0.522
**Distant metastasis**									
No	35 (85.4)	37 (72.5)		39 (76.5)	33 (80.5)		38 (76.0)	34 (81.0)	0.376
Yes	6 (14.6)	14 (27.5)	0.109	12 (23.5)	8 (19.5)	0.419	12 (24.0)	8 (19.0)	
**Clinical stage**									
1–2	30 (73.2)	19 (37.3)		28 (54.9)	21 (51.2)		29 (47.1)	20 (47.6)	
3–4	11 (26.8)	32 (62.7)	<0.001	23 (45.1)	20 (48.8)	0.044	21 (42.0)	22 (52.4)	0.021
**Germline BRCA mutation**									
No	8 (19.5)	10 (19.6)		9 (17.6)	9 (22.0)		7 (14.0)	11 (26.2)	
Yes	21 (51.2)	24 (47.1)	0.789	25 (49)	20 (48.8)	0.573	25 (50.0)	20 (47.6)	0.138
Unknown	12 (29.3)	17 (33.3)		17 (33.3)	12 (29.2)		18 (36.0)	11 (26.2)	
**Ki67**									
<20	3 (7.3)	7 (13.7)		3 (5.9)	7 (17.1)		2 (4.0)	8 (19.0)	
20–50	6 (14.6)	10 (19.6)	0.211	10 (19.6)	6 (14.6)	0.221	11 (22.0)	5 (11.9)	0.159
>50	32 (78)	34 (66.7)		38 (74.5)	28 (68.3)		37 (74.0)	29 (69.0)	

**Table 2:** Relationship between Trop-2, CD47, and CD163 expression levels.

	Trop-2 Expression			
	Negative/Low	High	*p*	*r*
**CD47 expression**	*n*, (%)	*n*, (%)		
Negative/Low	28 (68.3)	23 (45.1)	0.022	0.232
High	13 (31.7)	28 (54.9)		
**CD163 expression**				
Negative/Low	27 (65.9)	23 (45.1)	0.037	0.207
High	14 (34.1)	28 (54.9)		
	**CD47 Expression**			
	Negative/Low	High	*p*	*r*
**CD163 expression**	*n*, (%)	*n*, (%)		
Negative/Low	36 (70.6)	14 (34.1)	<0.001	0.364
High	15 (29.4)	27 (65.9)		

**Table 3:** The univariate Cox regression analysis of survival in patients with TNBC.

Univariate Analysis							
Overall Survival				Progression-Free Survival			
	HR	95.0% CI	*p*		HR	95.0% CI	*p*
Age	0.470	0.21–1.06	0.068	Age	0.450	0.20–1.01	0.054
Localization	1.140	0.52–2.50	0.737	Localization	1.130	0.52–2.48	0.754
Menopausal status	1.660	0.72–3.81	0.235	Menopausal status	1.700	0.74–3.92	0.211
ECOG PS	2.220	0.83–5.91	0.111	ECOG PS	2.210	0.83–5.90	0.112
Comorbidity	1.530	0.71–3.32	0.276	Comorbidity	1.460	0.68–3.16	0.337
Histological grade	1.130	0.56–2.26	0.736	Histological grade	1.120	0.56–2.23	0.758
Lymphovascular invasion	2.540	1.14–5.62	0.022	Lymphovascular invasion	2.550	1.16–5.64	0.021
Tumor focality	2.490	0.94–6.61	0.048	Tumor focality	2.860	1.07–7.62	0.036
Germline BRCA mutation	1.820	1.0–3.31	0.048	Germline BRCA mutation	1.930	1.06–3.52	0.031
pT stage	2.430	1.05–5.61	0.038	pT stage	2.480	1.07–5.75	0.035
Nodal involvement	1.950	0.87–4.40	0.105	Nodal involvement	1.940	0.86–4.35	0.109
Distant metastasis	4.470	1.96–10.2	<0.001	Distant metastasis	4.500	2.14–11.65	<0.001
Clinical stage	2.010	1.32–3.07	0.001	Clinical stage	2.080	1.35–3.19	0.001
Ki67	0.830	0.49–1.39	0.480	Ki67	0.830	0.49–1.38	0.465
CD47	0.670	0.30–1.51	0.335	CD47	0.810	0.37–1.77	0.596
CD163	0.800	0.37–1.74	0.573	CD163	1.210	0.74–1.98	0.439
Trop-2	7.980	2.70–23.52	0.001	Trop-2	8.540	2.87–25.37	0.001

**Table 4:** The multivariate Cox regression analysis of survival in patients with TNBC.

Multivariate Analysis							
Overall Survival				Progression-Free Survival			
	HR	95.0% CI	*p*		HR	95.0% CI	*p*
Lymphovascular invasion	2.880	1.24–6.73	0.014	Lymphovascular invasion	2.230	0.94–5.29	0.070
pT stage	2.280	0.90–5.78	0.083	pT stage	1.890	0.73–4.90	0.191
Distant metastasis	2.250	0.83–6.14	0.113	Distant metastasis	3.420	1.11–10.57	0.033
Clinical stage	1.120	0.66–1.89	0.680	Clinical stage	1.120	0.63–1.96	0.705
Germline BRCA mutation	1.820	1.0–3.31	0.048	Germline BRCA mutation	1.930	1.06–3.52	0.031
Tumor focality	1.120	0.32–4.20	0.527	Tumor focality	1.750	0.59–5.17	0.310
Trop-2 expression levels	8.320	2.69–25.68	<0.001	Trop-2 expression levels	9.940	3.13–31.53	<0.001

## Data Availability

The datasets used in this study can be made available by the corresponding author upon reasonable request, with permission from the Pathology Department of Cumhuriyet University School of Medicine.
